# Evaluating an Evidence-Based Parenting Intervention Among Filipino Parents: Protocol for a Pilot Randomized Controlled Trial

**DOI:** 10.2196/21867

**Published:** 2022-02-17

**Authors:** Samantha Reyes Macam, Wendy Mack, Lawrence Palinkas, Michele Kipke, Joyce Rivera Javier

**Affiliations:** 1 Fielding School of Public Health University of California Los Angeles Los Angeles, CA United States; 2 Department of Social Welfare Luskin School of Public Affairs University of California Los Angeles Los Angeles, CA United States; 3 Department of Population and Public Health Sciences Keck School of Medicine University of Southern California Los Angeles, CA United States; 4 Suzanne Dworak-Peck School of Social Work University of Southern California Los Angeles, CA United States; 5 Department of Pediatrics Keck School of Medicine University of Southern California Los Angeles, CA United States; 6 Division of Research on Child, Youth, and Families Children's Hospital Los Angeles Los Angeles, CA United States; 7 Division of General Pediatrics Children's Hospital Los Angeles Los Angeles, CA United States

**Keywords:** Filipino, mental health, prevention, parenting practices, community health

## Abstract

**Background:**

Filipino Americans underuse mental health and preventive care services even though studies have indicated that Filipino youth experience high rates of suicidal ideation, substance abuse, and teen pregnancies, whereas adults experience immigration stress, discrimination, and depression. Evidence-based parenting interventions provided in early childhood have proven to be effective in preventing the onset and escalation of child mental health disorders. In a pilot randomized controlled trial, we found that participation in the Incredible Years Basic Parent Training Program (IY) improved parenting stress and positive parenting practices and decreased child internalizing and externalizing symptoms among Filipino families. A fully powered trial is needed to determine the efficacy of IY as a prevention program among Filipino families.

**Objective:**

The aims of this study are to describe the design and rationale of a randomized controlled trial evaluating the effects of the web-based IY program among parents recruited from multiple community-based settings and its impact on parenting practices, parenting stress, and child problem behavior among Filipino Americans and describe the impact of COVID-19 on our study protocols.

**Methods:**

This study uses a randomized controlled 2-arm individually randomized group treatment pretest–posttest design for 180 parent–child dyads. Individuals are eligible if they are ≥18 years, live in California, and have at least one Filipino child aged 8-12 years. Consenting participants are randomly allocated to receive either the 12-week IY parenting intervention (intervention arm) or American Academy of Pediatrics’ Bright Future handouts and placed on a waitlist to receive IY posttrial (waitlist control arm). Primary outcomes include the Parent Practices Interview and the Parenting Stress Index. Secondary outcomes will be measured using the Child Behavior Checklist (completed by parent) and will include child internalizing and externalizing behaviors and total problems. Data are collected at baseline and 3- and 6-month follow-ups.

**Results:**

Changes made to the protocol owing to COVID-19 include administration of surveys remotely and implementation of the intervention on the web. The pandemic has provided an opportunity to evaluate the effectiveness of a web-based version of IY that may improve access and increase use of the intervention. Recruitment and data collection procedures are still ongoing and are expected to be completed by December 2022.

**Conclusions:**

Our research will determine whether IY promotes positive parenting practices and prevents child internalizing and externalizing behaviors in healthy but high-risk populations such as Filipino families. It will also uplift cultural narratives and add to the evidence base for web-based parenting programs and their implementation in real-world settings. If found efficacious, IY has the potential to prevent behavioral health disparities in this understudied and high-risk Filipino population and can be scaled, adapted, and implemented in other at-risk racial and ethnic minority communities.

**Trial Registration:**

ClinicalTrials.gov NCT04031170; https://clinicaltrials.gov/ct2/show/NCT04031170

**International Registered Report Identifier (IRRID):**

DERR1-10.2196/21867

## Introduction

### Filipino Mental Health

Filipino Americans are the third largest Asian American subgroup in the United States, with the highest concentration living in Los Angeles, California [1]. Despite their numbers, Filipinos have been described as an *invisible minority* and remain understudied owing to the lack of disaggregated research regarding their health status and needs [1-3]. In contrast to the *model minority myth* ascribed to Asians in general [4,5], US-born Filipino youth exhibit higher rates of mental health problems than non-Hispanic White youth [6]. The few studies on Filipino youth reveal exceedingly high rates of adolescent suicidal ideation and attempts compared with non-Hispanic White, Latinx, and African American individuals [2,7-9]. Filipino youth also have higher rates of behavioral problems such as conduct disorder, substance use, high-school dropout, and teen births than other Asian subgroups and have higher rates of depressive symptoms compared with non-Hispanic White individuals [2,8,10,11].

Filipino families also face acculturative and intergenerational challenges, parental separation owing to immigration, family conflict, parental mental health disorders, child maltreatment, loss of social status, discrimination, and high rates of major and postpartum depression [12-14]. The COVID-19 pandemic has exacerbated these issues and placed Filipino children at risk for future behavioral and mental health problems by increasing familial stressors and disrupting many avenues to access health services [15-17]. It has also revealed gaps in our existing approach to the prevention of perinatal depression and child mental health issues.

Despite these behavioral problems, Filipinos are less likely than non-Hispanic White individuals to participate in mental health care and preventive care use, including engagement in parenting interventions [10,18-20]. Barriers to participation include cultural stigma associated with parenting and mental health and time constraints among parents who often work multiple jobs [2,7,9,11,19]. For Filipinos, 85% of whom are affiliated with the Catholic Church [21], the clergy play a critical role in their efforts to manage personal problems [22,23] and, thus, have the potential to serve as gatekeepers to behavioral health and preventive services, including parenting programs. Schools, primary care providers, and community-based organizations may also serve as intervention gatekeepers. Conducting clinical trials in familiar and geographically accessible real-world settings may reduce some of the psychological and logistical barriers to participation and increase motivation to participate [24].

### Evidence-Based Parenting Programs

Evidence-based parenting interventions in early childhood have proven to be effective in preventing the onset and escalation of child mental and behavioral health disorders [25]. Many parenting programs target suboptimal parent–child relationships and harsh discipline, two critical risk factors for behavioral health problems among youth [26,27]. Parenting practices strongly affect child behavior problems, perhaps even playing a causal role [28]. The relationship between the antisocial traits of parents and their children is mediated in part by specific parenting practice [29]. There is a great deal of evidence that parenting has a powerful effect on improving functioning and reducing impairments [30]. Child behavior also affects parenting in a transactional manner [31-34]. Children’s challenging behaviors (eg, high activity level and poor emotion regulation, attention, and impulse control skills) can elicit coercive or detached parenting, with low nurturance and affection [35]. This parenting style may lead to an exacerbation of the child’s behavior problems [28,30,36-39].

In contrast, parental affection, supervision, and firm behavioral control predict long-term positive outcomes [37,40-43]. Parent training programs alter parents’ behavior and, presumably in response, children’s behavior [44,45]. The training program we are implementing prevents challenging child behaviors early and acts to interrupt this dysfunctional coercive cycle before the child’s behaviors become entrenched into an identifiable impairment and eligible for standard treatment models [46,47].

### Incredible Years School Age Advance and Basic Parent Training Program

Incredible Years Basic Parent Training Program (IY) is one of the best-studied and highly regarded parent training programs, with previous research highlighting the efficacy of the School Age Basic Parenting Training Program in the Filipino community [19,48-50]. The IY Advance Training Program includes topics focused on effective communication skills and based on community feedback, was suggested to be offered to Filipino families. Current evidence suggests that parent behavior-management programs such as IY are a viable treatment for reducing depressive symptoms in young children [51,52]. Over the past 11 years, Javier et al have conducted a series of studies to pinpoint parent training as a community-identified solution to prevent Filipino adolescent behavioral health problems and disparities [49]; pilot-test IY to assess the efficacy, feasibility, and acceptability of this prevention program in community settings in the Filipino population [50]; and develop a theory-based motivational video to increase Filipino parent enrollment rates in IY [53].

The success of these pilots underscores our ability to build trust with community organizations serving large Filipino populations and to overcome logistical challenges involved in implementing the intervention. Previous research conducted by project investigators justify the need to prevent behavioral problems among Filipino youth [2,10,54]. A prevention trial offering the IY School Age Basic and Advance Parent Training Program is needed to determine whether this 12-week combined intervention improves positive parenting practices and internalizing symptoms in children at higher risk for future depression, such as Filipinos.

### Theoretical or Conceptual Model

**Figure 1:**
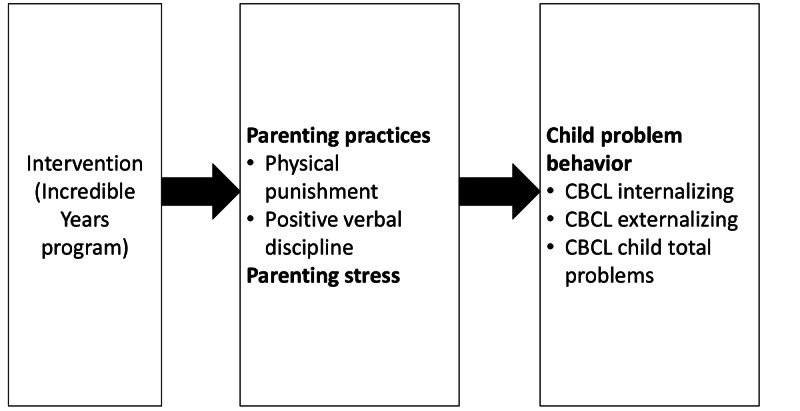
Conceptual model of Incredible Years program theory. CBCL: Child Behavior Checklist.

The conceptual model of the IY program in Figure 1 depicts the study hypotheses, that is, how IY affects parenting practices, parenting stress (intermediate outcomes), and child problem behavior (long-term outcomes).

Social learning theory suggests that early childhood internalizing symptoms may have roots in dysfunctional parenting behaviors and family environments [55]. For instance, depressive behaviors may be both modeled and selectively reinforced by parents [56]. IY targets some of the mechanisms and risk factors for internalizing distress in early childhood: harsh and unpredictable or critical parenting behaviors [57,58]. Parents also learn cognitive strategies for themselves, such as self-praise, coping thoughts, how to challenge negative thoughts, and how to get support that they are encouraged to model for and teach their children. Finally, they learn how to be more positive and nurturing through academic, social, and emotional coaching.

The knowledge gained from this trial will contribute to the scientific literature on preventive and early intervention programs for children at risk for future behavioral problems and to the literature on implementing evidence-based parenting interventions in real-world settings. Very few proven interventions that target Filipino parents are currently available. The data will also provide important information to understand the processes underlying the effects of IY on parenting practices and subsequent child problem behavior among Filipino families. The importance of this research rests on its potential to prevent behavioral health disparities in this understudied and high-risk population.

### Hypotheses

The purpose of this study is to describe the design and rationale of a randomized controlled trial evaluating the effects of a 12-week prevention program that includes content from the School Age Advance and Basic Parent Training Program on parenting practices, parenting stress, and child problem behavior among Filipino American families in a community-based setting. Our central hypothesis, based on social learning theory, is that IY will provide efficient parent training, resulting in significant improvements in parenting practices, parenting stress, and child behavioral problems among the intervention group compared with the control group. This hypothesis is supported by a previous study in which we determined initial estimates of effect sizes attributable to the intervention [50].

We hypothesize that parents will report improvements in positive parenting practices, parenting stress, and child problem behavior after IY as compared with baseline and the control condition.

## Methods

### Overview

This pilot trial is funded by grants from the Robert Wood Johnson Foundation Clinical Scholars Program and the University of Southern California Keck School of Medicine COVID-19 and Bridge Funding Program. Trial enrollment was initiated in July 2018 and is ongoing.

### Ethics Approval

This study was approved by the Children’s Hospital of Los Angeles Institutional Review Board (Study ID CHLA-18-00066).

### Trial Population

#### Inclusion and Exclusion Criteria

An individual is eligible to participate if he or she is (1) is aged ≥18 years and (2) identifies as a parent of at least one Filipino child aged 8-12 years. The inclusion criteria for the 180 Filipino American children are as follows: (1) aged between 8 and 12 years and (2) identify as Filipino or half-Filipino. Exclusion criteria include the following: (1) parent plans to move out of California during the next 9 months, (2) parent does not speak English, and (3) parent has completed IY in the past.

We are only including individuals who can speak English because according to the US Census Bureau data for Historic Filipinotown, a large proportion of the Filipino households (80%) are fluent in English [1]. This is primarily owing to the effects of American colonialism in the Philippines, during which an English-only curriculum was implemented. As the majority of Filipinos in Southern California are foreign-born, they have been educated in these institutions in which they were able to learn English. In a previous IY study that offered both consent forms and surveys in Tagalog, 100% of participants chose to use English forms [48].

#### Outreach and Recruitment

We are outreaching to Filipino parents of children aged 8-12 years from community sites (ie, churches, schools, primary care programs, after-school programs, and community-based organizations) serving Filipino families in California. The sample includes US citizens, permanent residents, and newer immigrants to ensure diversity in the range of socioeconomic status and length of stay in the United States. Prospective study participants are recruited via (1) announcements at regularly scheduled events with parents; (2) mailed letters, which include an endorsement by the community partner, a description of the study, and contact information so that parents can call if they would like to participate; (3) snowball sampling techniques; (4) study website with promotional video; and (5) use of social media.

To address barriers to participation, we use a parent engagement intervention video to promote recruitment. A screening instrument is used to screen potential participants for eligibility and obtain information regarding reasons for refusing to participate in the study. We provide gift card incentives for each survey completed; parents receive US $40 per survey and children receive US $10 per survey. Demographic information, including income, educational level, profession, immigration status, and insurance status, is obtained at baseline. Flyers, unaddressed letters, and email descriptions of the study are provided to partnering organizations for their staff to distribute to Filipino families. Parent addresses or emails are not given to study personnel without parental consent.

Snowball sampling is a nonprobability sampling technique where existing study participants recruit future participants from among their acquaintances and colleagues. We will use this method if we are unable to recruit enough participants from community sites. In these cases, to assure that names are not provided to researchers without their permission, participants may be asked to talk to their friends about this project. Participants are also asked to give their friends our contact information if they wish to become a part of the project. A flyer may be given to the participant to pass on to other potential respondents. The flyer can be used to contact the research team if the referred person is interested in participating in the study. If an individual is interested in participating, eligibility is confirmed using the inclusion and exclusion criteria. We also have a study website where parents may view the video we developed to promote participation in IY.

Once a parent is found to meet the study criteria and expresses an interest in the study, he or she is provided with a detailed description of the study and informed consent is obtained before the administration of preintervention surveys. If families decline to participate, verbal permission is obtained to ask about demographic information and the reason for refusal. A database of stated reasons for refusing and demographics is anonymously maintained and used to aid future recruitment efforts and compare participants with study refusals.

### Intervention

#### Study Design

This pilot study is an individually randomized controlled group treatment trial involving 180 parents of children aged 8-12 years to test the effects of IY implemented in community settings on parenting practices, parenting stress, child behavioral symptoms, and child functional status. Parents are informed of the randomized designs as part of the recruitment and consent procedures. Parents are randomly assigned in a 1:1 allocation with blocked randomization into either the intervention or control arm, resulting in 90 parent–child dyads in both the intervention and control groups. The trial statistician (WM) created the randomization list (using SAS [version 9.4; SAS Institute, Inc]). Research staff conducting data entry and collection are blinded to each participant’s randomized assignment.

#### IY Structure

Before delivering IY in this population, the researchers asked community members to pinpoint health issues they wanted to address through the use of a community-academic partnership and a community advisory board composed of participating Filipino parents and community partners [22]. Together, they came up with the solution of using evidence-based parenting programs to improve child mental health and behavioral issues. The use of community partners allows for increased capacity to outreach to Filipino American families across California.

A total of 2 parent group leaders who have completed the certified training in IY are responsible for delivering the intervention and cofacilitating each parenting workshop. To ensure adequate understanding of Filipino culture and parenting styles, at least one facilitator for each group must identify as Filipino. To track IY participation, parent group leaders record attendance after each session. Parents attend sessions without their child, and each group typically includes up to 15 parents. During each session, the leaders provide education on parenting strategies, play videos illustrating effective and ineffective parenting, incorporate role-playing exercises, and facilitate discussion about participants’ own personal experiences, opinions, and thoughts related to the session’s content to enhance their parenting skills.

IY sessions are offered to parents in the intervention group immediately after randomization. There are 12 weekly intervention classes, 2 hours each, for IY. Session topics are shown in Table 1. Sessions 1-6 include topics from the basic program, and sessions 7-12 include topics from the advance program.

**Table 1:** Session schedule of the Incredible Years program.

Week	Topic
1	Welcome, parent goals, and parental attention
2	Special time or parental attention
3	Social, emotion, and persistence coaching
4	Effective praise
5	Tangible rewards
6	Rules, responsibilities, and routines
7	Clear limit setting and ignoring misbehavior
8	Listening attentively
9	Speaking up
10	Communicating more positively to oneself and others; part 1
11	Communicating more positively to oneself and others; part 2
12	Giving and getting support, graduation, and celebration

We offer make-up sessions for parents missing a weekly session immediately before the next week’s session. For example, if a parent misses week 1 session, they are invited to arrive 30 minutes early for week 2 session to go over last week’s materials and concepts. The rationale for this is to ensure that the parents are exposed to the materials and concepts as much as possible. Overall, the IY curriculum builds upon principles discussed during the previous weeks.

### Comparator

Immediately after randomization, control participants are emailed and mailed written parent education materials from the American Academy of Pediatrics Bright Futures program. These materials include general parenting advice with age group–specific tips on how parents can support their child’s development and social and academic success. Parents in the control group are placed on a 3-month waitlist for the IY and are offered the 12 sessions after they have completed the follow-up assessments and intervention group classes end.

### Outcomes

Our main hypothesis is that the IY will provide efficient parent training, resulting in significant improvements in parenting practices, parenting stress (primary outcomes), child problem behavior, and COVID-19–related stress (secondary outcomes).

To evaluate primary outcomes, we use the Parenting Practices Inventory [52] to assess parenting practices and physical punishment and the Parenting Stress Index–Short Form [59] to assess parenting stress. To evaluate secondary outcomes, we use the Child Behavior Checklist for Ages 6 to 18 [60]: Externalizing and Internalizing domains and Total Problems (Parent, Child). The reliability and validity of these measures have been described in the literature, with all measures previously validated in multicultural populations. Finally, owing to the COVID-19 pandemic, we also measure parental COVID-19–related stress using the Epidemic–Pandemic Impacts Inventory [61]. To assess consumer perspectives, we use parent satisfaction and participation data in IY evaluation surveys.

### Data Collection

#### Overview

**Figure 2:**
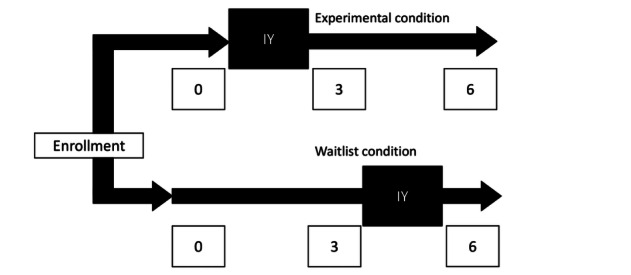
Data collection timeline. IY: Incredible Years Parent Training Program; 0: baseline assessment (wave 1); 3: 3-month follow-up (wave 2); 6: 6-month follow-up (wave 3).

Data collection occurs at three timepoints: at baseline, at 3-month follow-up, and at 6-month follow-up. Figure 2 provides a data collection timeline for both the intervention and waitlist control groups. Data are obtained using process evaluation tracking systems and parent- and child-report instruments.

Parents and children complete the assessments by answering the survey questions. During each interview, the interviewer reads the survey questions found in the assessment forms and records participant responses. The control group completes preintervention and postintervention assessments during the same period as the intervention group. Survey data are entered into a Research Electronic Data Capture trial database and Achenbach System of Empirically Based Assessment–Web databases. The method of data collection (in-person, phone, or web-based) and intervention delivery (in-person or on the internet) is recorded in the data set.

#### Baseline

After consent is obtained, outcomes assessments and demographic information, including income, educational level, profession, immigration status, insurance status, and levels of enculturation, are gathered for all participants at baseline. Assessments are scored immediately following the group that week and entered into the database.

#### 3-Month Follow-up

After the intervention group’s 12-week IY program ends 3 months after baseline, all participants including the control group complete postintervention questionnaires.

#### 6-Month Follow-up

All participants complete another postintervention questionnaire 6 months after baseline. This time frame also marks the end of the IY classes for control group participants, so the control group are asked to complete evaluations pertaining to their experience in IY.

### Protocol Changes Owing to COVID-19

Before the pandemic, all parenting sessions were held at various community-based settings. Owing to restrictions on in-person activities, the research team adapted the IY program to be held over Zoom, a web-based video conferencing platform that is compliant with the Health Insurance Portability and Accountability Act of 1996. Group leaders use presentation slides, Zoom chat functioning, discussion synopsis, breakout groups, and educational video clips, to ensure appropriate delivery of session material in a web-based format.

In addition, all surveys were previously administered in person at Children’s Hospital, Los Angeles. During the COVID-19 outbreak, all surveys were transitioned to be temporarily conducted over the phone or Zoom. Telephone and video surveys are administered by the interviewer in a quiet and private location, and participants are asked to take the call in a place that maintains their privacy.

We provide the option to use video call interviews for survey administration over a Health Insurance Portability and Accountability Act-compliant version of Zoom. The purpose of using Zoom is to ease the fatigue and comprehension of parents by allowing participants to read the survey questions via screenshare capabilities. Participants may have the option to remotely control entering their own answers if internet capabilities allow for remote control during screensharing. Interviewers screenshare the web-based Research Electronic Data Capture survey form and allow participants to read survey questions and select their answers that appear on the screen. Interviewers remain on the web to set up the screenshare of the survey and answer any questions that come up. Both telephone and video interviews are temporary to adjust to social distancing recommendations owing to COVID-19.

Once precautionary measures regarding COVID-19 are no longer required, in-person surveys with answers written on paper can resume. At that time, the option to conduct surveys through a video or telephone call may be provided to 3- or 6-month follow-up surveys as an alternative to the in-person survey as a means of increasing retention.

### Statistical Analysis

#### Power and Sample Size

Data from our preliminary pilot study were used to estimate sample size for a comparison of intervention group means in clustered data (clustering within individual IY groups). We used a Cohen *d* effect size of 0.55, intraclass correlation of 0.04, and coefficient of variation of 0.16 in cluster (IY group) sizes. Given this is a pilot study, each primary outcome and all secondary outcomes will be tested at α=.05. Using PASS software, sample sizes of 72 in each group (total sample 144), with an estimated 9 separate IY groups with an average of 8 participants in each group and 2-sided α of .05 will achieve 82% power to detect the intervention group effect size of at least 0.55. Predicting an annual attrition rate of 20%, a full sample of 180 participants is required.

#### Process Evaluation

We will document participants’ and facilitators’ experiences to ensure the appropriateness and cultural relevance of the intervention. Parent satisfaction and evaluation are also measured as part of process evaluation after program participation. Qualitative methods, such as field notes are used to document and analyze the intervention’s implementation and acceptability in the target population.

#### Analysis Plan

Before beginning hypothesis testing, data will be screened using univariate analyses (eg, mean, SD, plausible range and value, skewness, and kurtosis), patterns of correlation and covariance, and checks for multicollinearity and singularity of variables. Transformation of variables will be conducted as needed to reduce the effects of valid outliers or violations of normality. Participants will be analyzed by their randomized intervention group, regardless of adherence. Randomized groups will be compared on baseline values of demographic factors and trial primary and secondary outcomes; standardized group differences will be computed for each baseline variable as a standardized assessment of the magnitude of group differences. In this individually randomized group treatment trial, mixed effects linear models will be used to test the IY intervention effect on trial outcomes, accounting for correlated trial outcomes owing to participants clustered within individual IY groups. Mixed model random effects include random intercepts at the individual IY group level and separate random error terms for the IY and control participants. The model incorporates heteroscedasticity between IY versus control participants, as the modeled variance in the IY condition incorporates both group- and individual-level variability and differs from that of the waitlist condition. Fixed effects include the primary independent variable of the randomized group (IY vs waitlist); model covariates will include the baseline value of the trial outcome and baseline factors that differ between randomized groups.

Given the COVID-19 pandemic, we will analyze data from in-person intervention delivery versus web-based delivery separately. As mentioned above, we added a measure of COVID-19–related stress, the Epidemic–Pandemic Impacts Inventory-II. The number of COVID-19–related experiences endorsed will be summed within domains (work or employment, home life, social activities and isolation, changes in emotions and physical health and infection, and positive change) as a cumulative risk index or using an in-person clustering method to identify unique profiles of experiences.

## Results

Recruitment and data collection procedures are ongoing and are expected to complete by December 2022; we plan to complete data analyses by June 2023. As of December 2021, in total 103 have completed data collection in person and 60 have completed data collection on the web.

Our central hypothesis is that IY will provide efficient parent training, resulting in significant improvements in parenting practices, parenting stress, and child behavioral problems. This hypothesis is supported by a previous study in which we determined the initial estimates of effect sizes attributable to the intervention [50]. Findings of the pilot study revealed that IY had a positive impact on parenting stress, positive verbal discipline, physical punishment, and parent perceptions of their child’s externalizing symptoms, internalizing symptoms, and number of problematic behaviors [50].

Once data analysis is complete, results will be disseminated to individual partnering organizations and study participants, summarizing the results of the study. In addition, we will invite partnering organizations to a community forum aimed at sharing the results of the study and obtaining community feedback. Findings will also be submitted to a peer-reviewed journal and presented at academic conferences.

This study will provide a knowledge base for the translation of specific family-focused behavioral interventions to real-world practice settings with greater emphasis on maximizing the available resources within the contexts of local care settings (primary care settings, churches, schools, and community social service agencies) to better meet the needs of multiple stakeholders.

## Discussion

### Challenges

A few challenges arose during the study, one major obstacle being the COVID-19 pandemic. Owing to restrictions, we transitioned from in-person intervention implementation and providing the parenting group sessions in community spaces to the current remote delivery of the intervention. We worked with our community advisory board to develop the web-based protocol, and overall, families found it to be acceptable on the web. Of note, we also used materials developed by the IY developer to transition the program to the web (ie, use of web-based evaluations, tips for parenting group leaders to engage families virtually, and use of midweek individual phone calls to follow up with parents). Data collection including informed consent procedures and survey administration also transitioned from in-person to phone or Zoom conferencing communications. Unexpectedly, through the use of web-based platforms during the pandemic, we have been able to expand our outreach to different families across California and reduce time and transportation barriers to participating in the IY intervention. This web-based format seems to be more acceptable to families as it has helped overcome barriers such as transportation, commuting time to sites where in-person delivery occurred, child care, and the need for social distancing given the COVID-19 pandemic.

Another obstacle we faced was trial delay associated with use of a true community–academic partnership with numerous community partners and organizations helping to address mental health disparities in our communities. Some community partners involved have their own institutional review board and research process, which adds time to the overall regulatory review and approval. Development of statewide partnerships also requires significant time and effort, particularly when working with multidisciplinary teams including school districts, primary care clinics, and other community organizations.

### Comparison With Previous Work

Our previous work piloting IY as a community-based prevention program in partnership with gatekeepers and our community advisory board has made it possible to overcome stigma and time-related barriers [19,50]. We were also able to reach a population of at-risk children who have never been diagnosed or referred for behavioral health treatment, thus embodying the goal of prevention and early intervention. Although multiple studies have used churches and other community-based settings as means of engaging underserved, minority populations in mental health and preventative health programs [49,50,54,62-67], to our knowledge, no effectiveness trials among Filipinos have evaluated the IY as a prevention program offered in such settings. Moreover, few culturally appropriate programs specific to Filipino families exist.

To the best of our knowledge, this will also be the first study to examine the efficacy of IY as a web-based prevention program used to improve parenting practices and internalizing symptoms in school-aged children. Many of the previous studies using IY were performed with clinically referred patients and in primary care settings [68-71]. If we are able to determine that web-based IY also improves internalizing symptoms, this single intervention may be considered for use with other ethnic groups as a universal prevention strategy for healthy populations at risk for multiple behavioral problems (both externalizing and internalizing disorders).

### Future Directions

Results should suggest ways to increase the population-level effectiveness of parenting programs for minority and immigrant populations, who could benefit from such programs but tend to participate at low rates. These strategies will be used to design a future implementation trial that is attentive to the needs and preferences of parents and community stakeholders. Such strategies are critical in alleviating and eradicating behavioral health disparities seen in these populations. In addition, by transporting and evaluating effective interventions to a variety of community settings, we can provide critical information to health policy makers and public health leaders focused on making decisions about whether these interventions should be sustained.

### Conclusions

The knowledge gained from this trial will contribute to the scientific literature on preventive and early intervention programs for children at high risk for future behavioral problems as well as on the implementation of evidence-based parenting interventions in real-world settings. Very few proven interventions that target Filipino parents are available. The data will also provide important information to understand the processes underlying how IY affects parenting practices and subsequent child problem behavior among Filipino families.

This research will impact the well-being of Filipino youth and society as a whole by expanding the cultural narratives and the evidence base supporting web-based parenting interventions during middle childhood to prevent behavioral health disparities. It will allow us to identify and resolve challenges involved in implementing a behavioral parenting intervention in a large and growing immigrant population. The real value of this project is that it attempts to bridge the differences between immigrant parents and youth growing up in the United States and can serve as a model for promoting mental health equity among other immigrant communities affected by behavioral health disparities.

## Appendix

Multimedia Appendix 1 Peer Review Report from the USC Keck School of Medicine COVID-19 and Bridge Funding Program: Reviewer 1.

Multimedia Appendix 2 Peer Review Report from the USC Keck School of Medicine COVID-19 and Bridge Funding Program: Reviewer 2.

## Abbreviations

IY: Incredible Years Basic Parent Training Program

## References

[1] Hoeffel E, Rastogi S, Ouk KM, Shahid H (2012). The Asian Population: 2010. 2010 Census Briefs.

[2] Javier JR, Huffman LC, Mendoza FS (2007). Filipino child health in the United States: do health and health care disparities exist?. Prev Chronic Dis.

[3] Nadal K, Monzones J, Fujii DE (2010). Filipino Americans and neuropsychology. The Neuropsychology of Asian Americans (Studies on Neuropsychology, Neurology and Cognition).

[4] Choi Y, Lahey B (2006). Testing the model minority stereotype: youth behaviors across racial and ethnic groups. Soc Serv Rev.

[5] Kim PY, Lee D (2014). Internalized model minority myth, Asian values, and help-seeking attitudes among Asian American students. Cultur Divers Ethnic Minor Psychol.

[6] Buenavista TL (2010). Issues affecting U.S. Filipino student access to postsecondary education: a critical race theory perspective. J Educ Stud Placed Risk.

[7] Kuroki Y (2015). Risk factors for suicidal behaviors among Filipino Americans: a data mining approach. Am J Orthopsychiatry.

[8] Maramba D (2013). Family and educational environments: Contexts and counterstories of Filipino Americans. Educating Asian Americans: Achievement, Schooling, and Identities (Research on the Education of Asian Pacific Americans).

[9] Wolf DL (2016). Family secrets: transnational struggles among children of Filipino immigrants. Sociol Perspect.

[10] Javier J, Lahiff M, Ferrer R, Huffman L (2010). Examining depressive symptoms and use of counseling in the past year among Filipino and non-Hispanic white adolescents in California. J Dev Behav Pediatr.

[11] Willgerodt MA, Thompson EA (2006). Ethnic and generational influences on emotional distress and risk behaviors among Chinese and Filipino American adolescents. Res Nurs Health.

[12] Hayes DK, Ta VM, Hurwitz EL, Mitchell-Box KM, Fuddy LJ (2010). Disparities in self-reported postpartum depression among Asian, Hawaiian, and Pacific Islander Women in Hawaii: Pregnancy Risk Assessment Monitoring System (PRAMS), 2004-2007. Matern Child Health J.

[13] Kim HJ, Park E, Storr CL, Tran K, Juon H (2015). Depression among Asian-American adults in the community: systematic review and meta-analysis. PLoS One.

[14] Ying Y, Han M (2008). Parental acculturation, parental involvement, intergenerational relationship and adolescent outcomes in immigrant Filipino American families. J Immigr Refug Stud.

[15] Brown SM, Doom JR, Lechuga-Peña S, Watamura SE, Koppels T (2020). Stress and parenting during the global COVID-19 pandemic. Child Abuse Negl.

[16] Brunier A, Drysdale C (2020). COVID-19 disrupting mental health services in most countries, WHO survey. World Health Organization.

[17] Shah SM, Mohammad D, Qureshi MF, Abbas MZ, Aleem S (2021). Prevalence, psychological responses and associated correlates of depression, anxiety and stress in a global population, during the coronavirus disease (COVID-19) pandemic. Community Ment Health J.

[18] David EJ (2010). Cultural mistrust and mental health help-seeking attitudes among Filipino Americans. Asian Am J Psychol.

[19] Flores N, Supan J, Kreutzer CB, Samson A, Coffey DM, Javier JR (2015). Prevention of Filipino youth behavioral health disparities: identifying barriers and facilitators to participating in "Incredible Years," an evidence-based parenting intervention, Los Angeles, California, 2012. Prev Chronic Dis.

[20] Yu SM, Huang ZJ, Singh GK (2010). Health status and health services access and utilization among Chinese, Filipino, Japanese, Korean, South Asian, and Vietnamese children in California. Am J Public Health.

[21] Marineau M, Tice AD, Taylor-Garcia D, Akinaka KT, Lusk H, Ona F (2007). Culturally sensitive strategies designed to target the silent epidemic of hepatitis B in a Filipino community. Hawaii Med J.

[22] Javier JR, Supan J, Lansang A, Beyer W,  Kubicek K, Palinkas LA (2014). Preventing Filipino mental health disparities: Perspectives from adolescents, caregivers, providers, and advocates. Asian American Journal of Psychology.

[23] Nadal K (2011). Filipino American Psychology: A Handbook of Theory, Research, and Clinical Practice.

[24] Cunningham CE, Bremner R, Boyle M (1995). Large group community-based parenting programs for families of preschoolers at risk for disruptive behaviour disorders: utilization, cost effectiveness, and outcome. J Child Psychol Psychiatry.

[25] Kato N, Yanagawa T, Fujiwara T, Morawska A (2015). Prevalence of children’s mental health problems and the effectiveness of population-level family interventions. J Epidemiol.

[26] Mackenbach JD, Ringoot AP, van der Ende J, Verhulst FC, Jaddoe VWV, Hofman A, Jansen PW, Tiemeier HW (2014). Exploring the relation of harsh parental discipline with child emotional and behavioral problems by using multiple informants. The generation R study. PLoS One.

[27] Ackard DM, Neumark-Sztainer D, Story M, Perry C (2006). Parent-child connectedness and behavioral and emotional health among adolescents. Am J Prev Med.

[28] Patterson GR (1998). Continuities—A search for causal mechanisms: Comment on the special section. Developmental Psychology.

[29] Patterson G, Dishion T, Hinde RA, Stevenson-Hinde J (1998). Multilevel family process models: traits, interactions, and relationships. Relationships within Families: Mutual Influences.

[30] Chronis AM, Chacko A, Fabiano GA, Wymbs BT, Pelham Jr WE (2004). Enhancements to the behavioral parent training paradigm for families of children with ADHD: review and future directions. Clin Child Fam Psychol Rev.

[31] Bates JE, Pettit GS, Dodge KA, Ridge B (1998). Interaction of temperamental resistance to control and restrictive parenting in the development of externalizing behavior. Dev Psychol.

[32] Belsky J, Hsieh K H, Crnic K (1998). Mothering, fathering, and infant negativity as antecedents of boys' externalizing problems and inhibition at age 3 years: differential susceptibility to rearing experience?. Dev Psychopathol.

[33] Kochanska G (1995). Children's temperament, mothers' discipline, and security of attachment: multiple pathways to emerging internalization. Child Dev.

[34] Kochanska G (1997). Multiple pathways to conscience for children with different temperaments: From toddlerhood to age 5. Developmental Psychology.

[35] O'Connor TG, Deater-Deckard K, Fulker D, Rutter M, Plomin R (1998). Genotype–environment correlations in late childhood and early adolescence: antisocial behavioral problems and coercive parenting. Dev Psychol.

[36] Brennan PA, Le Brocque R, Hammen C (2003). Maternal depression, parent-child relationships, and resilient outcomes in adolescence. J Am Acad Child Adolesc Psychiatry.

[37] Galambos NL, Barker ET, Almeida DM (2003). Parents do matter: trajectories of change in externalizing and internalizing problems in early adolescence. Child Dev.

[38] Loeber R, Lösel F, Hurrelmann K (1990). Disruptive and antisocial behavior in childhood and adolescence: development and risk factors. Health Hazards in Adolescence.

[39] Stormshak EA, Bierman KL, McMahon RJ, Lengua LJ (2000). Parenting practices and child disruptive behavior problems in early elementary school. J Clin Child Psychol.

[40] Patterson GR, Chamberlain P (1994). A functional analysis of resistance during parent training therapy. Clin Psychol Sci Pract.

[41] Patterson GR, Forgatch MS (1995). Predicting future clinical adjustment from treatment outcome and process variables. Psychol Assess.

[42] Reid M, Webster-Stratton C, Beauchaine T (2001). Parent training in head start: a comparison of program response among African American, Asian American, Caucasian, and Hispanic mothers. Prev Sci Off J Soc Prev Res.

[43] Tolan PH, Gorman-Smith D, Henry DB (2003). The developmental ecology of urban males' youth violence. Dev Psychol.

[44] Brestan EV, Eyberg SM (1998). Effective psychosocial treatments of conduct-disordered children and adolescents: 29 years, 82 studies, and 5,272 kids. J Clin Child Psychol.

[45] Serketich WJ, Dumas JE (1996). The effectiveness of behavioral parent training to modify antisocial behavior in children: a meta-analysis. Behav Ther.

[46] Jensen PS, Hoagwood K, Petti T (1996). Outcomes of mental health care for children and adolescents: II. Literature review and application of a comprehensive model. J Am Acad Child Adolesc Psychiatry.

[47] Kazdin AE (1991). Effectiveness of psychotherapy with children and adolescents. J Consult Clin Psychol.

[48] Javier JR (2015). The Filipino family initiative: preliminary effects of an evidence-based parenting intervention offered in churches on parent and child outcomes. ProQuest.

[49] Javier JR, Supan J, Lansang A, Beyer W, Kubicek K, Palinkas LA (2014). Preventing Filipino mental health disparities: perspectives from adolescents, caregivers, providers, and advocates. Asian Am J Psychol.

[50] Javier JR, Coffey DM, Schrager SM, Palinkas LA, Miranda J (2016). Parenting intervention for prevention of behavioral problems in elementary school-age Filipino-American children: a pilot study in churches. J Dev Behav Pediatr.

[51] Herman KC, Borden LA, Reinke WM, Webster-Stratton C (2011). The impact of the Incredible Years parent, child, and teacher training programs on children's co-occurring internalizing symptoms. Sch Psychol Q.

[52] Webster-Stratton C, Herman KC (2008). The impact of parent behavior-management training on child depressive symptoms. J Couns Psychol.

[53] Javier J, Coffey D, Palinkas L, Kipke M, Miranda J, Schrager S (2019). Promoting enrollment in parenting programs among a Filipino population: a randomized trial. Pediatrics.

[54] Javier J, Chamberlain L, Rivera K, Gonzalez S, Mendoza F, Huffman L (2010). Lessons learned from a community-academic partnership addressing adolescent pregnancy prevention in Filipino American families. Prog Community Health Partnersh.

[55] Bandura A (1985). Social Foundations of Thought and Action: A Social Cognitive Theory.

[56] Hawkins W, Clarke G, Seeley J (1993). Application of social learning theory to the primary prevention of depression in adolescents. Health Values J Health Behav Educ Promot.

[57] Nelson JR, Stage S, Duppong-Hurley K, Synhorst L, Epstein MH (2016). Risk factors predictive of the problem behavior of children at risk for emotional and behavioral disorders. Except Child.

[58] Scaini S, Palmieri S, Caputi M (2018). The relationship between parenting and internalizing problems in childhood. IntechOpen.

[59] Abidin R (2012). Parenting Stress Index, fourth edition short form. PSI-4-SF.

[60] Achenbach T, Kreutzer JS, DeLuca J, Caplan B (2011). Child Behavior Checklist. Encyclopedia of Clinical Neuropsychology.

[61] Grasso D, Briggs-Gowan M, Ford J, Carter A (2020). Epidemic-Pandemic Impacts Inventory (EPII). UConn Health - Department of Psychiatry.

[62] Aguilar DE, Abesamis-Mendoza N, Ursua R, Divino LA, Cadag K, Gavin NP (2010). Lessons learned and challenges in building a Filipino health coalition. Health Promot Pract.

[63] Kataoka S, Fuentes S, O'Donoghue VP, Castillo-Campos P, Bonilla A, Halsey K, Avila JL, Wells KB (2006). A community participatory research partnership: the development of a faith-based intervention for children exposed to violence. Ethn Dis.

[64] Maxwell AE, Bastani R, Vida P, Warda US (2003). Results of a randomized trial to increase breast and cervical cancer screening among Filipino American women. Prevent Med.

[65] Maxwell AE, Bastani R, Danao LL, Antonio C, Garcia GM, Crespi CM (2010). Results of a community-based randomized trial to increase colorectal cancer screening among Filipino Americans. Am J Public Health.

[66] Ursua R, Aguilar D, Wyatt L, Katigbak C, Islam NS, Tandon SD, Nur PR, Van Devanter N, Rey MJ, Trinh-Shevrin C (2014). A community health worker intervention to improve management of hypertension among Filipino Americans in New York and New Jersey: a pilot study. Ethn Dis.

[67] Wells KB, Jones L, Chung B, Dixon EL, Tang L, Gilmore J, Sherbourne C, Ngo VK, Ong MK, Stockdale S, Ramos E, Belin TR, Miranda J (2013). Community-partnered cluster-randomized comparative effectiveness trial of community engagement and planning or resources for services to address depression disparities. J Gen Intern Med.

[68] Carson MC, Montaño Z, Kelman AR, Coffey DM, Javier JR (2019). Promoting behavioral health equity through implementation of the Incredible Years within primary care. Transl Issues Psychol Sci.

[69] Jones K, Daley D, Hutchings J, Bywater T, Eames C (2008). Efficacy of the Incredible Years Programme as an early intervention for children with conduct problems and ADHD: long-term follow-up. Child Care Health Dev.

[70] McGilloway S, Mhaille GN, Bywater T, Furlong M, Leckey Y, Kelly P, Comiskey C, Donnelly M (2012). A parenting intervention for childhood behavioral problems: a randomized controlled trial in disadvantaged community-based settings. J Consult Clin Psychol.

[71] Perrin EC, Sheldrick RC, McMenamy JM, Henson BS, Carter AS (2014). Improving parenting skills for families of young children in pediatric settings: a randomized clinical trial. JAMA Pediatr.

