# Supplementary material for: Evaluating an Evidence-Based Parenting Intervention Among Filipino Parents: Protocol for a Pilot Randomized Controlled Trial
Source: JMIR Res Protoc. 2022 Feb 17;11(2):e21867. doi: 10.2196/21867 (PMC8895283; doi:10.2196/21867)
Supplement: Multimedia Appendix 1 [file resprot_v11i2e21867_app1.pdf]

Reviewer 1 Scoring Sheet (Community)

Please complete this review form and be sure to click submit.

Thank you!

Response was added on 15/08/2020 11:51am.

[redacted]

General Instructions:

Score this proposal using the scoring system below. For each rated category, provide a few sentences/comments explaining your score. These may be shared with the applicant. Then provide an overall score using the same 1-9 rating system. Finally, you may provide confidential comments if you wish. These will be shared ONLY with the review committee and NOT with the applicant. Be sure to click SUBMIT when done.

Project Information Principal Investigator's Name Project Titles
Joyce Javier Preventing Child Maltreatment during COVID-19 Using On-line Evidence-based Parenting Interventions

SCORING SCALE Impact Score Descriptor Additional Guidelines on Strengths/Weaknesses
High 1 Exceptional Exceptionally strong with essentially no weaknesses
2 Outstanding Extremely Strong with negligible weaknesses
3 Excellent Very strong with only some minor weaknesses
Medium 4 Very Good Strong but with numersous minor weaknesses
5 Good Strong but with at least one moderate weakness
6 Satisfactory Some strengths but also some moderate weaknesses
Low 7 Fair Some strengths but with at least one major weakness
8 Marginal A few strengths and a few major weaknesses
9 Poor Very few strengths and numerous weaknesses
Minor Weakness: An easily addressable weakness that does not substantially lessen impact.

Moderate Weakness: A weakness that lessens impact.

Major Weakness: A weakness that severely limits impact.

Significance

Significance: major emphasis will be placed on the potential to inform communities and foster behaviors that will mitigate the pandemic and/or its consequences

[redacted]

2) Significance Comments

Interventions are needed to encourage positive parenting among Filipino families to prevent child abuse and internalizing/externalizing problems among children. During COVID-19 (and after), online interventions will be especially needed. This application will test the feasibility and acceptability of adapting an evidence-based parenting program for online delivery among Filipino families. This intervention has the potential to improve family functioning during and after the pandemic.

Investigators

Investigators: qualifications, of PI and/or team to develop, deliver and evaluate the impact of the proposed education and/or engagement; for collaborations outside of the Keck School, the value added of the collaborators and costs to support that element of the project available from other school(s)

[redacted]

4) Investigators Comments

Dr. Javier has been developing and implementing parenting programs for Filipino families for nearly two decades. She has considerable expertise in this area and has excellent relationships with community partners. She is well prepared to conduct this project.

Innovation

Innovation: Innovation will be valued primarily in the context of the potential impact on the community(ties) proposed for engagement and/or education

[redacted]

6) Innovation Comments

This study is somewhat incremental because it is an adaptation of an existing program, but the transformation of an in-person program to online is innovative and timely.

Approach

Approach

1. The content or structure of the proposed education or engagement

2. Target community/population, including known risks and impact of the COVID-19 impact

3. Methods for delivery, including match to target community(ties)

4. Approach to evaluation of impact

5. Next steps, if any

[redacted]

8)	Approach Comments	The approach is strong. The PI has clarified that this will be a feasibility trial that will generate an effect size estimate for a fully-powered randomized controlled trial. The adaptation of the program for the online implementation has already begun. The PI has obtained community input and will continue to do so throughout the process. The study design and statistical analyses are strong.

Environment

Environment

[redacted]

10)	Environment Comments	The environment is ideal to conduct this research. All necessary infrastructure is in place.

Overall Impact

[redacted]

12)	Overall Impact Comments	This study will make an excellent public health impact on the Filipino community during a stressful time. The PI has addressed the reviewers' previous concerns.

Confidential Comments to Executive Advisory Committee
