# Supplementary material for: Evaluating an Evidence-Based Parenting Intervention Among Filipino Parents: Protocol for a Pilot Randomized Controlled Trial
Source: JMIR Res Protoc. 2022 Feb 17;11(2):e21867. doi: 10.2196/21867 (PMC8895283; doi:10.2196/21867)
Supplement: Multimedia Appendix 2 [file resprot_v11i2e21867_app2.pdf]

Reviewer 2 Scoring Sheet (Community)

Please complete this review form and be sure to click submit.

Thank you!

Response was added on 18/08/2020 3:00pm.

[redacted]

General Instructions:

Score this proposal using the scoring system below. For each rated category, provide a few sentences/comments explaining your score. These may be shared with the applicant. Then provide an overall score using the same 1-9 rating system. Finally, you may provide confidential comments if you wish. These will be shared ONLY with the review committee and NOT with the applicant. Be sure to click SUBMIT when done.

Project Information Principal Investigator's Name Project Titles
Joyce Javier Preventing Child Maltreatment during COVID-19 Using On-line Evidence-based Parenting Interventions

SCORING SCALE Impact Score Descriptor Additional Guidelines on Strengths/Weaknesses
High 1 Exceptional Exceptionally strong with essentially no weaknesses
2 Outstanding Extremely Strong with negligible weaknesses
3 Excellent Very strong with only some minor weaknesses
Medium 4 Very Good Strong but with numersous minor weaknesses
5 Good Strong but with at least one moderate weakness
6 Satisfactory Some strengths but also some moderate weaknesses
Low 7 Fair Some strengths but with at least one major weakness
8 Marginal A few strengths and a few major weaknesses
9 Poor Very few strengths and numerous weaknesses
Minor Weakness: An easily addressable weakness that does not substantially lessen impact.

Moderate Weakness: A weakness that lessens impact.

Major Weakness: A weakness that severely limits impact.

Significance

[redacted]

2)	Significance Comments	This is a important pilot study that will assess the ability to transition an evidence-based intervention (IY) to an on-line platform to meet the parenting needs of families. This pilot is quite small, and while power calculations are provided, it is unclear, particularly given the reach the PIs appear to have, why this pilot would not be more robust. While all of IY works on strengthening parent-child interactions and attachment, reducing harsh discipline and helping parents promote children's social, emotional, and academic development, this program states it focuses on parental monitoring, rules setting and regulation, and how to support homework. These are particularly relevant as children are learning virtually, yet none of these outcomes is measured. It may be something for the PIs to consider.

Investigators

Investigators: qualifications, of PI and/or team to develop, deliver and evaluate the impact of the proposed education and/or engagement; for collaborations outside of the Keck School, the value added of the collaborators and costs to support that element of the project available from other school(s)

[redacted]

4)	Investigators Comments	Strong team that has history working together. Have prior experience implementing IY intervention (in person) with Filipino parents.

Innovation

Innovation: Innovation will be valued primarily in the context of the potential impact on the community(ties) proposed for engagement and/or education

[redacted]

6)	Innovation Comments	Given the current climate in which families are particularly stressed, the ability to adapt an evidenced-based parenting intervention is critical. The PIs speak to using this as pilot data for a larger federally funded project which would allow for a more robust trial

Approach

Approach

1. The content or structure of the proposed education or engagement

2. Target community/population, including known risks and impact of the COVID-19 impact

3. Methods for delivery, including match to target community(ties)

4. Approach to evaluation of impact

5. Next steps, if any

[redacted]

8)	Approach Comments	The additions made by PIs increases clarity related to recruitment, enrollment, and other procedures. A concern of this reviewer is retention. This is an incredible challenging time for families and a 12-week intervention is long. There is no room to "lose" anyone enrolled given the sample size, yet retention is not addressed. How many sessions must a family complete to get enough "dose". What will be done if families drop out? Will further recruitment occur?<br><br>Further unclear is the qualitative aspect of this project. The authors propose to complete a fairly straightforward evaluation of parent satisfaction; why it would be analyzed using grounded theory is unclear. Further, the CAB appears to be used for the intervention adaptation, and the interviews with parents post intervention. That data would not be merged in any meaningful way. This lends to further confusion about why the CAB meeting would be transcribed and analyzed in this way. Both of these elements are important, but are really traditional aspects of implementation science (for which grounded theory is not traditionally used).

Environment

Environment

[redacted]

10)	Environment Comments	Strong environment; well established in the community. Virtual intervention increases accessibility for all families

Overall Impact

[redacted]

12)	Overall Impact Comments	This is an important topic (telehealth adaptation of an EBI) and assessing a significant current need. PIs have a well established relationship and prior experience with implementation (with this group) in a traditional on-ground format. Given family stressors during the pandemic, parents could benefit from the support and learning. however, the stress of the pandemic also raises questions related to retention (12 weeks may add strain to families) and more clarity on approach (particularly why a more traditional implementation science approach is not being use) is needed.

Confidential Comments to Executive Advisory Committee
